# Relationship between regional wall shear stress and carotid plaque composition using 3 T MRI and patient-specific computational fluid dynamics

**DOI:** 10.1186/1532-429X-11-S1-O44

**Published:** 2009-01-28

**Authors:** Raymond Q Migrino, Mark Bowers, Leanne Harmann, Robert Prost, Anil Doppalapudi, Tayyab Mohyuddin, Megan Bright, Jason Jurva, Osama Zaidat, John LaDisa

**Affiliations:** 1 Medical College of Wisconsin, Milwaukee, WI USA; 2 Marquette University, Milwaukee, WI USA

**Keywords:** Computational Fluid Dynamic, Wall Shear Stress, Carotid Plaque, Computational Fluid Dynamic Model, Necrotic Core

## Introduction

Plaque vulnerability arises from the interplay among factors including plaque composition (PC) and wall shear stress (WSS). To date, the relationship between spatial WSS and PC is not established.

## Purpose

Our aim is to determine the relationship between WSS and PC in established carotid atherosclerosis.

## Methods

5 subjects (4 males, 66 ± 8 years), with moderate to severe carotid plaque underwent 3 T MRI using 4-channel carotid coil. T1, T2, proton density and time of flight images were obtained (0.47–0.55 × 0.47–0.55 × 2 mm spatial resolution) 12 mm above and below the bifurcation. Plaque composition (necrotic core and loose matrix) were quantified using Plaqueview software (VP Diagnostics). Subject-specific computational fluid dynamic models were created from MRI, B-mode ultrasound and blood pressure (BP) data using CVsim software. Outlet boundary conditions that replicated flow and BP were applied and simulations used a stabilized finite element solver. Each carotid slice were divided into 6 circumferential regions where WSS was correlated with PC.

## Results

**Figure 1:**
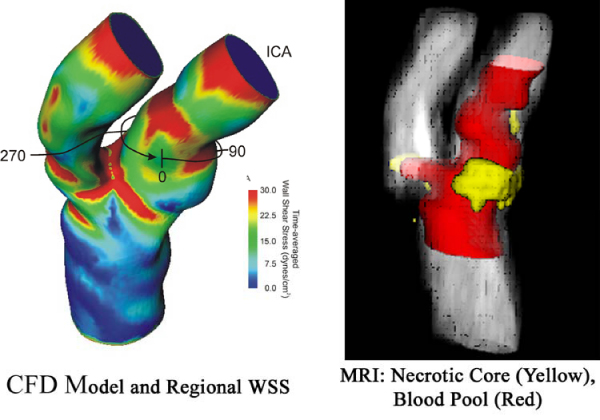
Figure 1

Please see figure 1. WSS correlated significantly with necrotic core (R = 0.283, p < 0.001) but not with loose matrix (R = -0.03, p = 0.6). The same relationship was seen in the common carotid, bifurcation or internal carotid artery. Carotid plaque regions with necrotic core had higher WSS than those without (34.1 ± 2.6 vs. 17.3 ± 4.6 dyn/cm^2^, p < 0.001). WSS in regions with and without loose matrix did not differ (25.8 ± 3.6 vs. 22.5 ± 3.3, p = 0.7).

## Conclusion

In established carotid artery disease, regions with high WSS are associated with increased necrotic core, but not loose matrix. This relationship between increased WSS and necrotic core content in the plaque may play an important role in plaque vulnerability.

